# Spontaneous Breathing and Pendelluft in Patients with Acute Lung Injury: A Narrative Review

**DOI:** 10.3390/jcm11247449

**Published:** 2022-12-15

**Authors:** Po-Lan Su, Zhanqi Zhao, Yen-Fen Ko, Chang-Wen Chen, Kuo-Sheng Cheng

**Affiliations:** 1 Department of Biomedical Engineering, National Cheng Kung University, Tainan 704, Taiwan; 2 Department of Internal Medicine, National Cheng Kung University Hospital, College of Medicine, National Cheng Kung University, Tainan 704, Taiwan; 3 Department of Biomedical Engineering, Fourth Military Medical University, Xi’an 710032, China; 4 Institute of Technical Medicine, Furtwangen University, 78120 VS-Schwenningen, Germany; 5 College of Biomedical Engineering, China Medical University, Taichung 404, Taiwan

**Keywords:** acute respiratory distress syndrome, spontaneous breathing, pendelluft

## Abstract

Acute respiratory distress syndrome (ARDS) is characterized by acute-onset rapid-deteriorating inflammatory lung injury. Although the preservation of spontaneous breathing may have physiological benefits in oxygenation, increasing evidence shows that vigorous spontaneous breathing may aggravate lung injury (i.e., patient self-inflicted lung injury). Increased lung stress and pendelluft, which is defined as intrapulmonary gas redistribution without a significant change in tidal volume, are important mechanisms of patient self-inflicted lung injury. The presence of pendelluft may be considered a surrogate marker of vigorous inspiratory effort, which can cause the dependent lung to overstretch. In this review, we summarized three major methods for electrical impedance tomography–based pendelluft monitoring. Future studies are warranted to compare and validate the different methods of pendelluft estimation in patients with ARDS.

## 1. Introduction

Acute respiratory distress syndrome (ARDS), which is characterized by acute-onset rapid-deteriorating inflammatory lung injury, affects approximately 10% of patients in the intensive care unit (ICU) [1]. Pathologically, it is characterized by extensive alveolar inflammation, increased vascular permeability, increased lung weight, and decreased aeration area [2]. The preservation of gentle spontaneous breathing during ARDS may have physiological benefits in oxygenation owing to the improved aeration of the dependent lung [3] and the redistribution of pulmonary blood flow toward the nondependent lung [4]. In addition, the maintenance of optimal diaphragm muscle contraction reduces the risk of ventilator-induced diaphragm dysfunction (VIDD) [5,6]. However, increasing evidence shows that spontaneous breathing may aggravate lung injury. In the present review, we summarize the beneficial and harmful effects of spontaneous breathing effort and also introduce pendelluft as a surrogate marker of vigorous breathing effort.

## 2. The Beneficial Effects of Spontaneous Breathing

### 2.1. Prevention of Diaphragm Dysfunction

In 2004, Vassilakopoulos and Petrof [7] first introduced VIDD, which is defined as decreased diaphragm muscle contractility secondary to the use of mechanical ventilation. The incidence of VIDD in patients who received mechanical ventilation ranges from 23% to 84% [8]. Diaphragm contractile activity decreases with increasing ventilator driving pressure and controlled ventilator mode [6]. To avoid the disuse atrophy of the diaphragm during mechanical ventilation, the preservation of spontaneous breathing would provide longer ventilator-free days and a shorter ICU length of stay [9]. These data indicate that the maintenance of optimal spontaneous breathing effort could be considered a method for preventing diaphragm dysfunction [10].

### 2.2. Increased Ventilation in the Dependent Lung

During spontaneous breathing, inspiratory muscle effort, which is mainly caused by diaphragm muscle contraction, will cause pleural pressure to become more negative [11]. The lowering of the pleural pressure will expand the lung by increasing transpulmonary pressure. Among patients with ARDS receiving pressure support ventilation, lower support level with higher inspiratory effort was associated with increased ventilation toward the dependent lung [12]. The difference in ventilation distribution could be explained by the cephalad movement of diaphragm in controlled ventilation, thus leading the ventilation to the nondependent region. By contrast, the dependent part of the diaphragm moves the most during spontaneous breathing, thus increasing the amount of ventilation to the dependent region [13]. Therefore, the maintenance of optimal spontaneous breathing effort may improve dependent lung ventilation and increase antero-posterior ventilation homogeneity and even oxygenation [12].

## 3. The Harmful Effects of Spontaneous Breathing—Patient Self-Inflicted Lung Injury (P-SILI)

### 3.1. Patient-Ventilator Asynchronies

Spontaneous breathing may occur in a vigorous form in patients with ARDS [14]. Protective lung ventilation remains the standard in patients with ARDS because pulmonary overdistention will further worsen the injured lung [15]. Breath stacking (double triggering) was found to occur frequently in nonparalyzed, sedated patients with acute lung injury under low tidal volume ventilation [16]. The number and duration of the cluster of double triggering and/or ineffective efforts are related to the duration of mechanical ventilation and ICU stay, as well as with hospital mortality in mechanically ventilated ICU patients [17]. The ACURASYS trial reported that breath stacking may offer one explanation for the beneficial effect of neuromuscular agent administration because breath stacking will not occur in paralyzed patients [18]. However, the Reevaluation of Systemic Early Neuromuscular Blockade trial reevaluated the benefits of cisatracurium in patients with ARDS and found that there was no difference in 90-day mortality when compared with sedation only [19]. Light sedation and higher positive end-expiratory pressure may be associated with less reverse triggering and less breath stacking [20]. The objective assessment of patient-ventilator asynchrony may provide the final answer.

### 3.2. Excessive Lung Perfusion

Vigorous inspiratory effort causes strong negative intrathoracic pressure, which results in excessive fluid shift from intrapulmonary vessels to interstitial and alveolar spaces [21,22]. This phenomenon has been described in patients with vigorous inspiratory effort against airway obstruction and is also called post-obstructive pulmonary edema [23]. In animal studies, abrupt deflation after sustained inflation, which is associated with a dramatic decrease in intrapulmonary pressure, could cause acute lung injury mediated by the significant vascular leakage and acute decompensation of the left ventricle [24,25]. These data imply that lung injury may also be caused by the abrupt and large change in intrathoracic pressure.

### 3.3. Increased Lung Stress and Pendelluft

The presence of a strong spontaneous breathing effort leads to a dramatic fluctuation in tidal volume. High tidal volume with high transpulmonary pressure would lead to lung injury [26]. Unfortunately, limiting tidal volume may not be safe. In an observational study of a patient with postoperative hypoxemia, high inspiratory effort caused early inflation in the dependent lung area and simultaneous deflation in the nondependent lung area [27]. The consolidative or atelectatic lung would attenuate the transmission of transpulmonary pressure and cause more local overdistension during diaphragm contraction [28]. This phenomenon was termed pendelluft, which is characterized by intrapulmonary gas redistribution without a significant change in tidal volume [27]. Pendelluft is clinically significant because it represents regional lung hyperdistention despite low tidal volume ventilation. The degree of pendelluft is proportional to the breathing effort even in reverse triggering [29]. However, a higher inspiratory effort is not always associated with a higher pendelluft volume [30]. Patients with high magnitude pendelluft also had increased inflammatory mediators related to acute lung injury independently of transpulmonary pressure [31]. Therefore, it would be better to monitor pendelluft and esophageal pressure simultaneously.

All three phenomena pertain to “P-SILI,” which is a lung injury caused by vigorous spontaneous breathing effort [32]. A high respiratory drive may also activate the expiratory muscles, thus resulting in negative expiratory transpulmonary pressure [22,33] and possibly causing subsequent lung derecruitment [22]. Asynchronies such as breath stacking and increased perfusion secondary to extreme effort may be easily assessed clinically, but pendelluft monitoring is difficult. The presence of pendelluft could only be assessed using electrical impedance tomography (EIT). In the following section, we introduce the three different methods of EIT-based pendelluft estimation.

## 4. Monitoring Pendelluft in Patients with Lung Injury

To accurately monitor intrapulmonary gas distribution, EIT, which allows for the non-invasive dynamic measurement of intrapulmonary ventilation distribution, has been implemented in many clinical and animal studies [34,35].

### 4.1. Estimation of Regional Lung Inflation during Spontaneous Breathing by Controlled Ventilation

**Figure 1:**
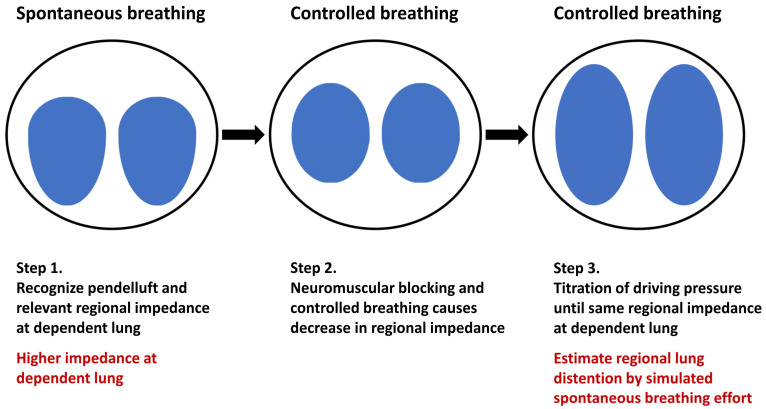
The estimation of the regional lung inflation of spontaneous breathing by controlled ventilation. During the whole estimation process, the regional ventilation of the patient is monitored by the EIT system (Pulmovista 500^®^, Draeger medical GmbH, Luebeck, Germany). At the beginning of spontaneous breathing, the strong inspiratory effort causes significant inflation and impedance change at the dependent lung region. After controlled breathing with neuromuscular blocking agents, the regional impedance changes at the dependent lung decreases. To estimate the regional lung inflation of the spontaneous breathing effort, incremental titration of the driving pressure is applied until the same regional impedance change at the dependent lung is achieved.

Yoshida et al. [27] first described a method for estimating the regional lung inflation during spontaneous breathing. In an animal study, the maximal impedance change in the dependent lung region at the beginning of inspiration was recorded when pendelluft developed. After the administration of the muscle relaxant, the driving pressure was continuously titrated until the same impedance change developed in the dependent lung (Figure 1). In their study, a significantly larger tidal volume was observed after achieving comparable impedance changes in the dependent lung, thus indicating overdistention in the dependent lung. Although this method could only estimate regional lung inflation instead of the amount of pendelluft, it could still provide information on regional lung stress. However, this method may use an extremely high driving pressure, which might increase the risk of ventilator-induced lung injury and limit its clinical application.

### 4.2. Estimation of Intrapulmonary Gas Flow

**Figure 2:**
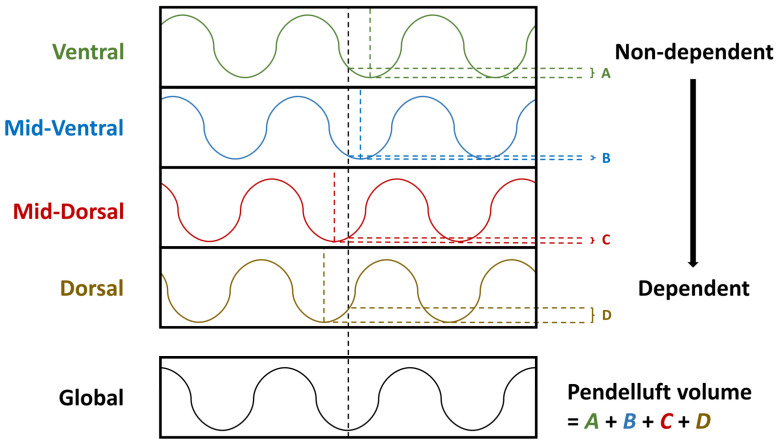
The quantitative analysis of pendelluft. The global impedance–time curve is presented in the bottom column and the timepoint of nadir impedance was recognized as the starting point of inspiration (black dash line). The impedance–time curve of each region of interest is presented in the upper column, and the timepoints of nadir impedance were also defined, including ventral (green dash line), mid-ventral (blue dash line), mid-dorsal (red dash line), and dorsal (brown dash line) parts. The impedance difference of each region between the global and regional nadir timepoints were calculated and summed as the pendelluft volume.

On the basis of this real-time monitoring device, Coppadoro et al. [34] proposed a pendelluft estimation method. In brief, global and regional impedance–time curves were analyzed. To evaluate intrapulmonary gas flow across the dependent and nondependent lung regions, the regions of interest can be classified from ventral to dorsal into four layers of the same thickness. The time point at the nadir of the global impedance was considered the initiation of inspiration (T0). Similarly, the time point of the nadirs of each layer was identified (Ti). Subsequently, the volume change of each region between the time point of the regional nadir (Ti) and global nadir (T0) was calculated (Figure 2) [30]. In their study, the amount of pendelluft increased significantly with decremental titration of pressure support ventilation in some of their patients. This method is practical for clinical use but has a drawback, namely, the assumption of a homogeneous lung zones in each layer, which may result in an underestimation of the pendelluft volume. The threshold of a high pendelluft and its correlation with regional lung stress should be validated in a future study.

### 4.3. Measurement of Pendelluft Amplitude

**Figure 3:**
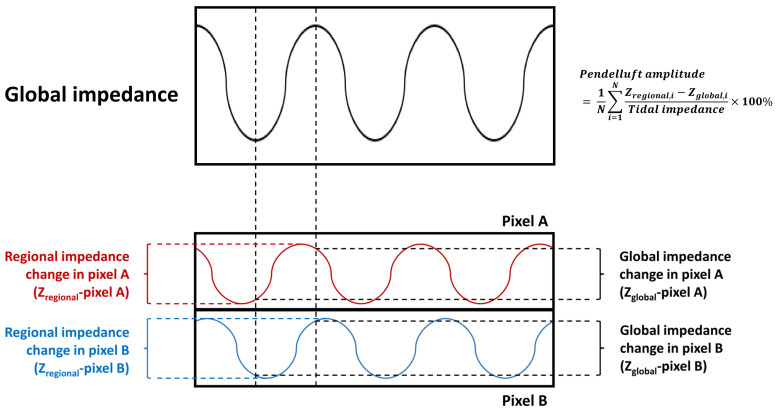
The estimation of pendelluft amplitude. The global impedance–time curve is presented in the upper column, and the timepoint of nadir impedance and peak impedance are recognized as the starting and ending points of inspiration (black dash line). The bottom column presents the impedance–time curve from two consecutive pixels. The regional impedance change of the pixel was defined by the impedance change in a breath cycle. Meanwhile, the global impedance change of the pixel was defined by the impedance difference in each pixel between the starting and ending timepoints of inspiration. The difference between regional and global impedance changes is summed as the pendelluft amplitude.

Recently, Sang et al. [36] introduced another estimation method based on electrical impedance tomography, which can measure the pendelluft for each pixel. First, the timepoints of nadir (T_nadir_) and peak (T_peak_) in each breathing cycle of the global impedance–time curve are identified as the starting time points of inspiration and expiration, respectively. The impedance differences regarding the global ventilation time point (Z_global_) were established by subtracting the impedance at T_nadir_ from that at T_peak_. Second, the regional impedance–time curve of each pixel was created. The impedance difference between the peak and nadir of each breathing cycle for each pixel is calculated (Z_regional_). If ventilation is homogenous during tidal breathing, the global impedance change (Z_global_) will be close to the regional impedance change (Z_regional_). The pendelluft amplitude is estimated using the following formula (Figure 3):

(1) $$Pendelluft\;amplitude=\frac{1}{N}\sum_{i=1}^{N}\frac{\left(Z_{regional,i}-Z_{global,i}\right)}{tidal\;volume}\times 100\%$$

Measurement errors and noise may also cause differences between Z_regional_ and Z_global_ values. A retrospective observational study that evaluated 30 healthy volunteers defined a pendelluft amplitude that is >2.5% of the tidal volume as a clinically significant pendelluft [37]. In a subsequent validation cohort of 200 patients with ARDS, a clinically significant pendelluft was associated with longer ventilator-dependent days in patients with severe hypoxemia (PaO_2_/FiO_2_ ratio < 200 mmHg) [35]. However, given that the threshold was defined on the basis of healthy volunteers, different cut-off points might have occurred in patients with ARDS.

## 5. Conclusions

Appropriate spontaneous breathing may prevent VIDD and improve ventilation homogeneity but may cause P-SILI when breathing effort is excessive. By using standard protective lung ventilation, patient-ventilator asynchronies and extreme effort can be easily detected. However, pendelluft could only be assessed by EIT monitoring. Future studies are warranted to compare and validate the different methods of pendelluft estimation in patients with ARDS.

## Data Availability

Not applicable.
